# Supplementary material for: LncRNA BCYRN1 as a Potential Therapeutic Target and Diagnostic Marker in Serum Exosomes in Bladder Cancer
Source: Int J Mol Sci. 2024 May 29;25(11):5955. doi: 10.3390/ijms25115955 (PMC11172611; doi:10.3390/ijms25115955)
Supplement: Supplementary file 1 [file ijms-25-05955-s001.zip › Supplemental Figures S1-S3.pdf]

Supplemental Figure S1

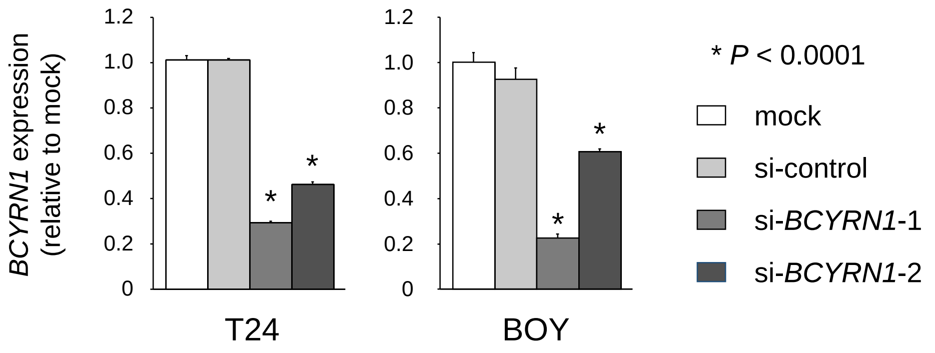

Figure S1: Knockdown efficiency of si*BCYRN1*-1/-2 for BC cell lines (T24 and BOY cells)

Supplemental Figure S2

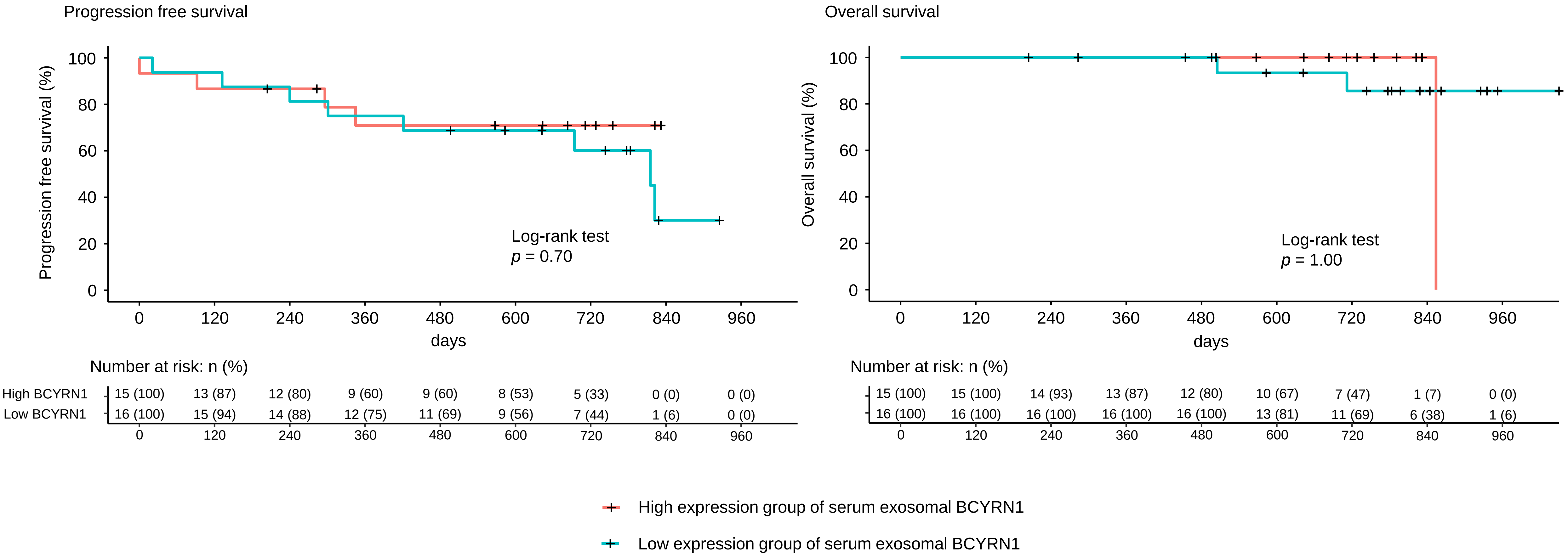

Figure S2: Kaplan-Meyer curve of progression-free survival for the *BCYRN1* high-expression group (solid line) and low-expression group (dashed line)

Supplemental Figure S3

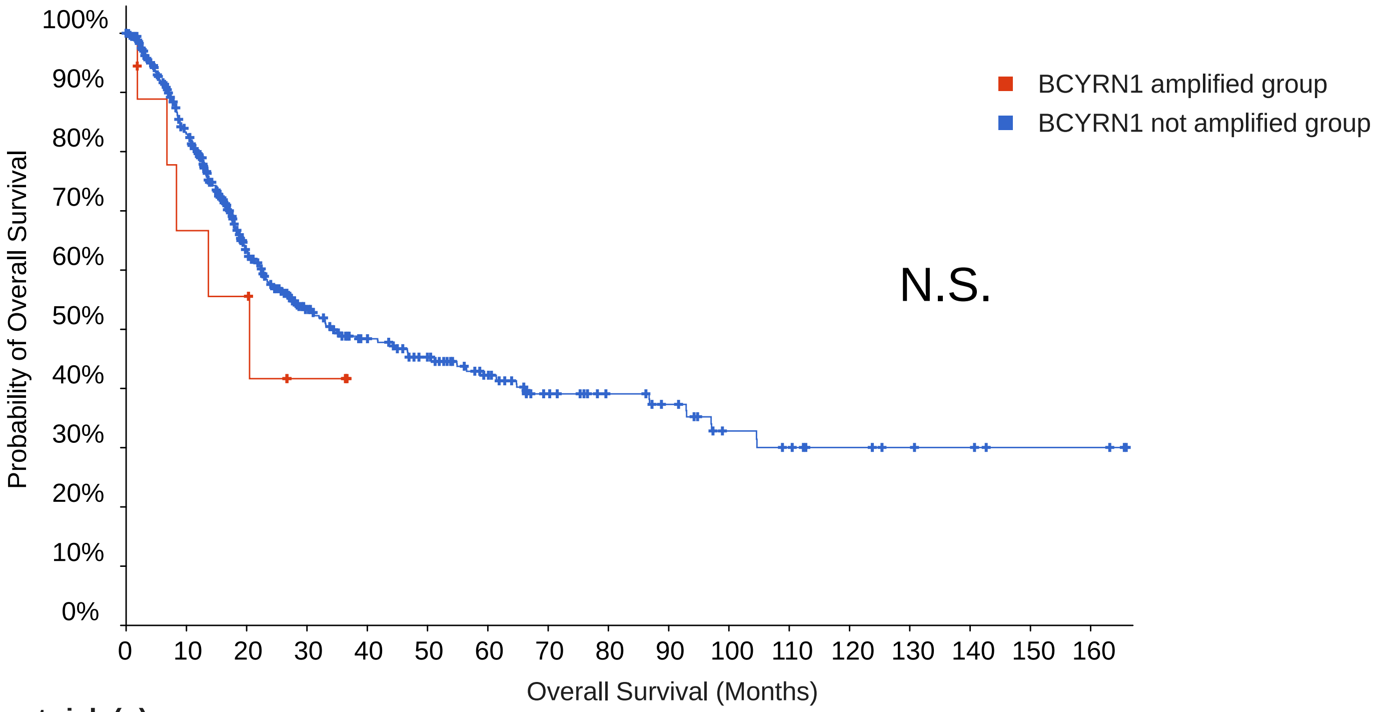

Number at risk (n)

Altered group	18	12	10	4	0	0	0	0	0	0	0	0	0	0	0	0	0
Unaltered group	1639	1236	665	420	322	266	186	118	91	75	47	39	27	22	17	12	12

Figure S3: Kaplan-Meyer curve of overall survival in groups in which *BCYRN1* was not amplified (blue) and was amplified (red)
